# Prolactin and Male Fertility: The Long and Short Feedback Regulation

**DOI:** 10.1155/2009/687259

**Published:** 2008-11-03

**Authors:** M. K. Gill-Sharma

**Affiliations:** Department of Neuroendocrinology, National Institute for Research in Reproductive Health, Jehangir Merwanji Street, Parel, Mumbai 400 012, India

## Abstract

In the last 20 years, a pituitary-hypothalamus tissue culture system with intact neural and portal connections has been developed in our lab and used to understand the feedback mechanisms that regulate the secretions of adenohypophyseal hormones and fertility of male rats. In the last decade, several in vivo rat models have also been developed in our lab with a view to substantiate the in vitro findings, in order to delineate the role of pituitary hormones in the regulation of fertility of male rats. These studies have relied on both surgical and pharmacological interventions to modulate the secretions of gonadotropins and testosterone. The interrelationship between the circadian release of reproductive hormones has also been ascertained in normal men. Our studies suggest that testosterone regulates the secretion of prolactin through a long feedback mechanism, which appears to have been conserved from rats to humans. These studies have filled in a major lacuna pertaining to the role of prolactin in male reproductive physiology by demonstrating the interdependence between testosterone and prolactin. Systemic levels of prolactin play a deterministic role in the mechanism of chromatin condensation during spermiogenesis.

## 1. Introduction

Male fertility in mammals is regulated by the two adenohypophyseal hormones, luteinizing hormone (LH) and follicle stimulating hormone (FSH), through synthesis of testosterone in the interstitial cells of Leydig and its aromatization to estradiol in the Sertoli cells of the testis. The regulated release of the hypothalamic gonadotropin-releasing hormone (GnRH) ensures normal functioning of the hypothalamo-hypophysio-gonadal axis, through secretion of gonadotropins and testosterone in systemic circulation, necessary for spermatogenesis, maturation of spermatozoa, and reproductive behaviour [1, 2]. Prolactin, a 23 Kd hormone, synthesized in the adenohypophyseal lactotrophs, has no known target organ or defined role in male reproduction. Yet, expression of prolactin receptors on choroid plexuses and hypothalamus presupposes a latent role for this hormone in the regulation of male fertility [3, 4]. In particular, advancement of knowledge in the area of prolactin signaling cascades across species suggests a conserved physiological role from rodents to humans [5]. Although the functional significance of prolactin to male reproduction has not been unequivocally established, the hormone has been associated primarily with male infertility. Acute hyperprolactinemia is known to suppress testosterone synthesis and male fertility through prolactin-induced hypersecretion of adrenal corticoids or by inhibiting the secretion of GnRH through prolactin receptors on hypothalamic dopaminergic neurons [6, 7]. Dopamine has been implicated in the release of endorphins from opiatergic neurons that inhibit the secretion of GnRH [8].

Since a clear consensus about the involvement of prolactin in male reproduction has not emerged even from genetically engineered mouse models wherein prolactin signaling has been disrupted, not enough attention has been focused on the regulation of its secretion [9, 10]. However, the absence of detrimental effects on male fertility in prolactin receptor-deficient mice does not preclude a role for prolactin, but rather indicates the existence of compensatory mechanisms reported in literature for certain genetically modified mouse models [11, 12]. Albeit, genetically modified mice overexpressing prolactin, could prove to be alternative models in understanding the physiological role of this hormone in modulating male fertility [13]. A host of releasing and release-inhibiting factors of pituitary and hypothalamic origin have been suggested to be involved in prolactin secretion but their physiological relevance in male reproduction remains a moot point. Dopamine remains the only acceptable negative regulator of prolactin secretion of physiological relevance [14]. Prolactin primarily regulates its own release via a short feedback loop through autoreceptors on the hypothalamic tuberoinfundibular dopaminergic neurons (TIDA), which release dopamine (DA) into the long portal vessels [15]. Dopamine reaches the adenohypophysis through pituitary portal circulation where its inhibitory effects are mediated via cognate D2 dopaminergic receptors on the lactotrophs [16]. Among the gonadal steroids, high estradiol levels appear to render the dopaminergic neurons refractory to prolactin autofeedback making it improbable to reverse the effects of physiological or pathological hyperprolactinemia [17–19]. In such an eventuality, it is tempting to assume that additional androgen-dependent feedback mechanisms might be involved in restraining the secretion of prolactin, which has a propensity for hypersecretion [20].

Conventionally, prolactin is without a target organ and, therefore, not subject to long feedback regulation. However, prolactin has also been reported to play a role in the synthesis of testosterone through upregulation of LH receptors on Leydig cells but the relevance of this mechanism to reproductive physiology has not been understood [21, 22]. Testosterone is known to be involved in the feedback inhibition of LH, the hormone responsible for its synthesis in the testis [23]. By analogy, it is again tempting to assume that a similar mechanism of feedback inhibition by testosterone could also exist for prolactin. Such a mechanism could conceivably be playing a vital role in male reproductive physiology by modulating prolactin and testosterone levels in systemic circulation under conditions of mild-to-moderate hyperprolactinemia arising out of emotional or chemical stress. Thus, in view of the doubtful significance of prolactin in the regulation of male fertility, lacunae continue to persist pertaining to mechanisms underlying its secretion. Towards this end, in vitro and in vivo models developed in our lab have focused on this aspect. Several novel findings about feedback mechanisms have emerged from these studies that suggest a modulatory role for prolactin in the regulation of male fertility.

## 2. Hypothalamo Hypophyseal-Gonadal Factors Underlying Prolactin Secretion

**Figure 1:**
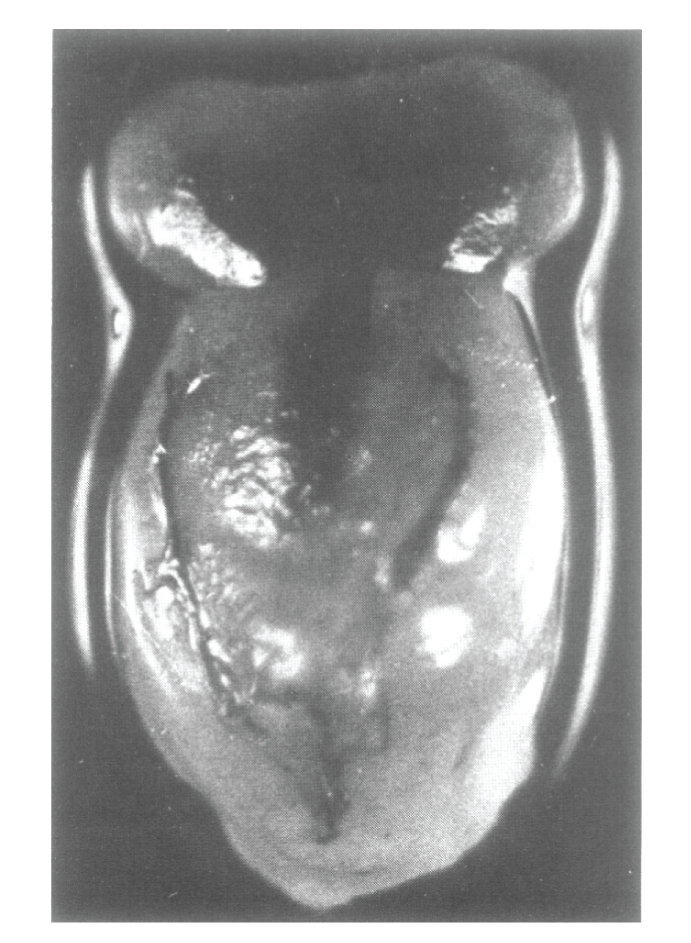
A photograph of a freshly dissected pituitary-hypothalamus complex (PHC) from a normal adult male rat killed 5 minutes, following intracardiac injection of India ink [24].

Prolactin secretion is regulated by factors originating from the hypothalamus, the hypophysis, and the testes in the male rats. These factors are either inhibitory or stimulatory in action such that their cumulative effect determines the systemic levels of this hormone. The hypothalamic input to the pituitary could be in the form of hypothalamic-inhibitory activity (HPIA) or hypothalamic-releasing activity (HPRA). The physiological relevance of inhibitory and stimulatory factors remains to be unequivocally demonstrated. Towards this end, we have used an in vitro organ culture system comprising the rat hypothalamus and pituitary in anatomic juxtaposition to establish the existence of complex interactions between putative factors from both organs. The development of the rat pituitary-hypothalamus tissue culture (PHc) with intact neural connections and portal plexus in our lab in 1985 was a major advancement in reproductive Neuroendocrinology that spawned several studies designed to understand the feedback regulation of pituitary hormones in vitro [24]. The hypothalamic island, demarcated by cutting along the lateral hypothalamic sulci, posterior edge of the optic chiasma, and the anterior edge of the mammillary bodies, was lifted from its seat on the Sella turcica with an undercut 2-3 mm in depth, along with the pituitary attached to it via hypophyseal stalk (see Figure 1). PHc comprises all the major dopaminergic perikarya viz. Tuberoinfundibular (TIDA), periventricular (PHDA), tuberohypophyseal (THDA), as well as the long and short portal vessels [25, 26]. PHc and intact pituitaries (PI) were incubated in Dulbecco's Modified Eagle's Medium (DMEM), fortified with nonessential amino acids, 3 mM NaHCO_3_ and 0.1 M HEPES (4-(2-hydroxyethyl)-piperazine-1-ethansulfonic) acid, and 0.1% bovine serum albumin at 37°C for periods that varied from 1–72 hours depending on the experiment. The hormones were assayed in the media, blood, or pituitary homogenates by radioimmunoassays. Surgical interventions like castration from 1–46 days, chemical treatments like in vitro exposure to testosterone, or pharmacological exposure to drugs in vivo were routinely employed to manipulate the hormonal status.

### 2.1. Hypothalamic Factors

Dutt et al. (1986) first used the adult rat PHc cultures as the method of choice to study the inhibitory influence of hypothalamus over the kinetics of secretion of prolactin from the adenohypophyseal lactotrophs in vitro [27]. The authors observed that intact pituitary released comparable amounts of prolactin from 2 to 6 hours in vitro. The hourly basal rate of prolactin release decreased from 2 to 6 hours of incubation (Table 1). Coincubation with the hypothalamus neither suppressed the amounts secreted nor altered the rate of pituitary prolactin secretion from 2–6 hours. The data was interpreted to suggest that the hypothalamic prolactin releasing factors (PRFs) were necessary to maintain the basal secretion of prolactin from the pituitary glands. The secretion of prolactin from PHc was observed to be considerably lower than that from intact pituitary or coincubates. The rate of prolactin release from PHc decreased from 4 to 6 hours. It could be inferred from the comparative kinetics of prolactin secretion from intact pituitary and PHc cultures that when the portal connections were maintained in vitro between the adeno- and neurohypophyses and the neural and portal connections maintained with the hypothalamus, the rate of prolactin secretion was more physiological because of the availability of the hypothalamic PRFs and PIFs. However, the functionality of the dopaminergic inhibitory receptors on the lactotrophs in the PHc culture model only became evident when these systems were challenged with a dose of 1 × 10^−7^ M dopamine in vitro. Only the PHc responded to the dopamine challenge and exhibited a decrease in the rate of prolactin secretion.

Dopamine has been shown to inhibit the release of prolactin in a variety of in vitro systems [28–32]. In another set of experiments designed to further validate PHc as a model system of choice to study prolactin release, Dutt et al. (1992) compared the effects of dopamine, its agonists and antagonists in PHc, whole pituitary (PI), adenohypophysis (P-N), P-N+PP (posterior pituitary) cultures from normal and reserpine-treated (prolactin depleted) rats [33]. The authors observed that as compared to the other systems, PHc, which released less prolactin than the other systems, also responded adequately to agonists like 10^−7^ M dopamine, 10^−7^ M Bromocriptine, 8 × 10^−7^ M Apomorphine with suppression, and antagonists like 5 × 10^−8^ M Perphenazine with stimulation of prolactin release, even after reserpine-induced depletion of lactotrophs (Table 2). The authors concluded that if the intact portal and neural connections between the anterior and posterior lobes of the pituitary and the hypothalamus were not maintained, the dopaminergic receptors on the lactotrophs no longer responded to dopamine challenge in vitro, since the latter hormone, if not stored in the posterior pituitary, got degraded rapidly [34]. It was inferred from these studies that prolactin had a characteristic pattern of release in vivo which was dependent on the delivery of hypothalamic PRFs and PIFs to the neurohypophysis through neural connections and reached the adenohypophysis through neurohypophyseal portal capillaries. Functional pituitary DA receptors were absolutely essential for the dopaminergic inhibitory mechanism to operate, both in vitro and in vivo, for maintaining homeostasis of prolactin.

**Figure 2:**
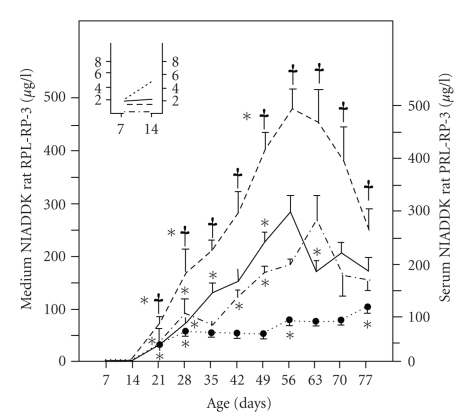
Age-related changes in serum concentrations of prolactin (dotted line), release of prolactin by whole pituitary (free release; dashed line), hypothalamic prolactin-releasing activity (solid line), and hypothalamic prolactin-inhibiting activity (dotted-dashed line) in male rats. Values are mean ± SEM from Values are mean ± SEM from two separate experiments and a minimum number of 14 animals per pooled group. *P < .05 compared with preceding group; ^†^ *P* < .05 compared with hypothalamic prolactin-releasing activity at the same age (Student's *t*-test). The inset shows the results for days 7 and 14 on an expanded scale. The pituitary constructs were dissected out of 7 to 77 day-old rats and incubated in DMEM for 4 hours at 37°C. Prolactin was estimated by RIA in the spent medium [35].

Having established the suitability of the PHc cultures for studying the regulation of prolactin secretion in vitro, Karanth et al. (1987) used the rat PHc and PI cultures to establish the ontogenetic pattern of maturation of the hypothalamic releasing and release-inhibiting activities that regulate its systemic levels at adulthood [35]. Several endogenous factors have been reported to stimulate prolactin secretion viz. Serotonin, *β* endorphin, Met enkephalin, Leu-enkephalin, thyrotropin-releasing hormone (TRH), luteinizing hormone releasing hormone (LHRH), substance P, vasopressin, oestradiol-17-*β*, epidermal growth factor, fibroblast growth factor, cholecystokinin, and angiotensin II, prolactin-releasing peptides (PrRPs) by direct action at the pituitary level [36–40]. To qualify as PRFs, these factors would have to override the inhibitory control of prolactin secretion by physiological PIFs like dopamine and GABA [41–43]. The prolactin released by the intact pituitary in vitro is a measure of unregulated release whereas that secreted by PHc in vitro is the cumulative effect of physiological PRFs. Dutt et al. (1986) assumed that since PHc secreted less prolactin than intact hypophysis in vitro, the difference in the amount released by the two systems would reflect the cumulative hypothalamic PIF activity (HPIA) whereas that secreted by PHc should be a true reflection of hypothalamic PRF activity [27]. Karanth et al. (1987) used PHc and PI cultures to validate this hypothesis. They attempted to correlate the developmental changes in the HPRA and HPIA activities in terms of prolactin released from PHc and PI in vitro from postnatal day 7 to 77, to the age-related changes in the serum prolactin levels in male rats, as reported in literature [35, 44]. The pituitary weights and prolactin content increased from day 7 to 70 but was maintained thereafter in adult rats. The weight of the hypothalamus increased from postnatal day 7 to 56 before stabilization at maturity. Serum prolactin levels underwent age-related increase from birth to 77 days with two peaks at days 49 and 77. The HPIA activity was undetectable in vitro prior to day 21, peaked from days 35 to 49, decreased at day 70, and was maintained thereafter. The HPRA activity increased in vitro through days 7 to 56, decreased on day 63, and stabilized (see Figure 2). The data was suggestive of the presence of potent PRF activity prior to age 21. The inference drawn from this study was supported by the findings of Becú & Libertun (1982) and Khorram et al. (1984) who reported potent age-related PRF activities viz. TRH activity on day 1 and serotonergic PRF activity on day 12 in male rats [45, 46]. Thus, the authors concurred that age-related increase in serum prolactin levels till day 28 could be attributed to the concomitant increase in HPRA. Since the serum prolactin levels peaked again at day 70, despite stabilization of HPIA and HPRA activities, it was inferred that extrahypothalamic factors like gonadal estradiol could be modulating the responsiveness of the pituitary to dopamine. Alternatively, changes in the sensitivity of pituitary to HPIA and HPRA could have led to increased secretion at adulthood. This conclusion was based on the prevailing hypothesis that the responsiveness of pituitary to TRH, LHRH, DA, and estradiol undergoes a change from puberty to adulthood [47–50]. It was inferred that the levels of prolactin in circulation were determined by competitive interactions between HPIA and HPRA, in accordance with the suggestion of Matsushita et al. [51–53].

### 2.2. Testicular Factors

**Figure 3:**
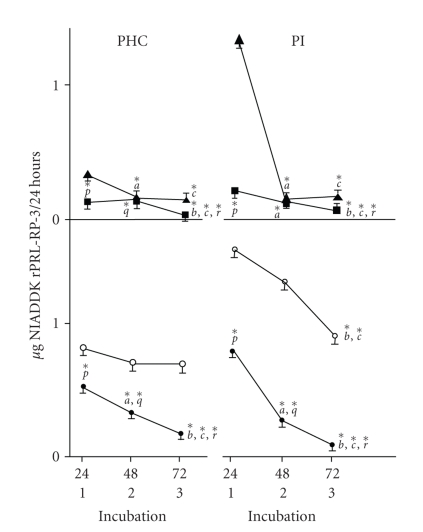
Release of PRL by PHC and PI of normal and castrated rats incubated with or without testosterone for 72 hours. Upper panel. Pattern of release of PRL by PHC and PI of castrated rats, incubated in absence (▴) or presence (▪) of testosterone (0.3 mM) for 72 hours. Values are mean ± SE and derived from 40 individual incubations of PHC and 16 of PI, respectively. Lower panel. Pattern of release of PRL by PHC and PI of normal rats incubated in absence (∘) or presence (•) of testosterone (0.3 mM) for 72 hours. Values are mean ± SE and derived from 15 individual incubations of PHC and 16 PI, respectively. *a*, *b*, *c* denote significant difference in values at 48 hours as compared to 24 hours, at 72 hours as compared to 48 hours, and at 72 hours as compared to 24 hours, respectively. *p*, *q*, *r* denote significant difference in groups incubated with or without testosterone at 24, 48, and 72 hours, respectively. *P ≤ .05. Pituitary constructs were dissected out of normal or 7-day castrated rats and incubated in DMEM with or without 0.3 mM testosterone, at 37°C for 24–72 hrs. Prolactin was analysed in the spent medium by RIA [54].

**Figure 4:**
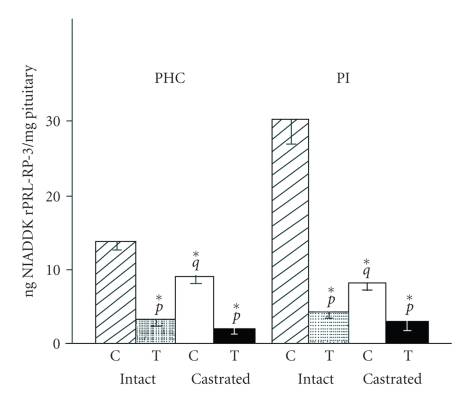
Intrapituitary contents of PRL after incubation of PHC (left-hand panel) and PI (right-hand panel) from normal rats without (stippled bars) or with (hatched bars) testosterone (0.3 mM) or from castrated rats incubated without (open bars) or with (solid bars) testosterone (0.3 mM) for 72 hours. Values represent mean ± SE derived from 10 pituitaries. *p*, *q*, *r*, denote significant differences in groups incubated with or without testosterone, normal controls versus castrated controls and between normal incubated with testosterone versus castrated incubated with testosterone, respectively. *P ≤ .05. C: Incubation without testosterone; T: Incubation with testosterone. Pituitary prolactin content was estimated by RIA in PBS homogenates of pituitary constructs incubated with or without 0.3 mM testosterone for 72 hours and a challenge dose of LHRH at 37°C for 4 hours [54].

Prolactin can upregulate the synthesis of testosterone in vivo through cognate receptors on the Leydig cells of the testis [21, 22]. Based on intuitive logic that end products are generally involved in the feedback regulation of their hormonal stimulus, Gill-Sharma et al. (1992) studied the inhibitory effects, if any of testosterone on prolactin secretion from rat PHc and intact pituitary of normal and 7-day castrated rats in vitro [54]. The authors could demonstrate the existence of a novel long loop feedback mechanism for the regulation of prolactin release in sexually competent male rats. They observed that temporal release of prolactin from PHc of normal rats was constant from 24 to 72 hours whereas that from PHc from castrated and intact pituitary of normal and castrated rats decreased from 24 to 72 hours of incubation in vitro (see Figure 3). The release rates per 24 hours from normal PHc constructs were significantly lower than those from intact pituitary constructs. The decrease in intact pituitary basal release rates could be attributed to absence of hypothalamic PRFs. The decrease in release from castrated PHc and intact pituitary constructs was likely due to either the lack of PRFs or availability of PIFs from the median eminence or both. Coincubation with testosterone suppressed the temporal secretion rate of prolactin from both PHc and intact pituitary constructs of normal and castrated rats from 24 to 72 hours in vitro. This observation was interpreted to suggest that testosterone primarily acted on the pituitary lactotrophs of both constructs to suppress prolactin secretion. Interestingly, prolactin content of incubated pituitaries from PHc constructs of normal rats was lower than that in intact pituitaries but this difference disappeared after castration. This observation was suggestive of suppressive effects of testosterone on prolactin synthesis. The inhibitory effect of testosterone became evident from the clear suppression of prolactin content both in incubated pituitaries from PHc and intact pituitaries of normal and castrated rats (see Figure 4). The functionality of the 72-hour incubated pituitary constructs was demonstrable when all pituitary constructs not exposed to testosterone, responded to a challenge dose of 100 pm LHRH over a 4-hour period, with enhanced release of LH and FSH, whereas androgen exposure counteracted this response. The underlying mechanism through which testosterone suppressed prolactin remains to be delineated at the biochemical level. It can be speculated that prolactin receptors expressed on the pituitary lactotrophs could have mediated this effect [55]. Prolactin has been reported to exert a proliferative effect on pituitary GH3 cells, expressing a cognate receptor [56]. Hyperplasia of the adenohypophysis is a hallmark of mice with dysfunctional prolactin signaling, akin to the effect observed in dopamine receptor D2-deficient mice, and suggestive of an antiproliferative role of hypothalamic dopamine in antagonizing the prolactin receptor-mediated growth response [14, 57]. It remains to be seen whether testosterone can exert an antiproliferative effect on lactotrophs by downregulating the prolactin receptor, observed in rat liver, to eventually reduce the level of prolactin [58].

Gill-Sharma et al. (2001) had reported that the suppression of LH induced by low doses of estradiol in male rats did not inhibit the secretion of testosterone presumably due to an effect of prolactin on its synthesis in the Leydig cells [59]. Testosterone inhibition however occurred at higher estradiol doses only after enhancement of systemic prolactin levels (see Table 3). Presumably, estradiol-induced enhancement of rat prolactin secretion in vivo occurs through estrogen response elements in its gene in the pituitary gland and leads to infertility [60].

**Figure 5:**
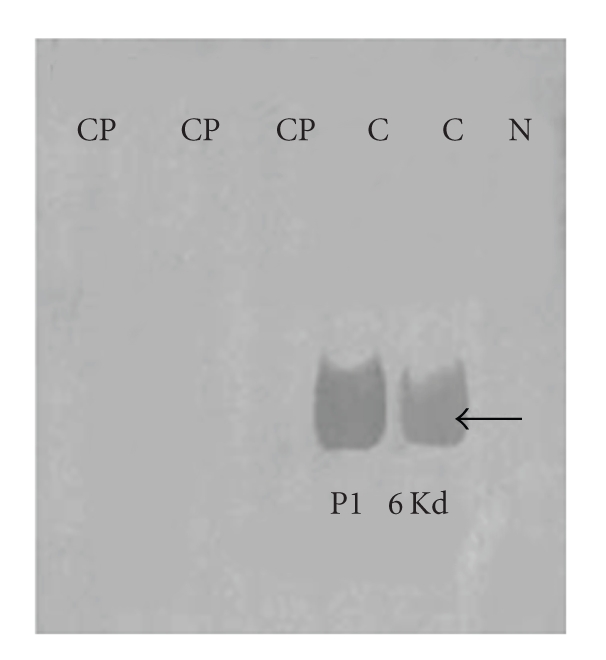
Western blots of protamine 1 in epididymal sperm of sexually mature rats depicting the presence of 6 Kd protamine 1 bands in control and absence in drug-treated rats. Cp: Cyproterone acetate; C: Control; N: negative control. Basic proteins were extracted in HCl, differentially extracted in trichloroacetic acid, analysed on 15% acid urea PAGE. Western blotting was done with 1Hup 1N monoclonal antibody provided by Dr. Rod Balhorn [61].

**Figure 6:**
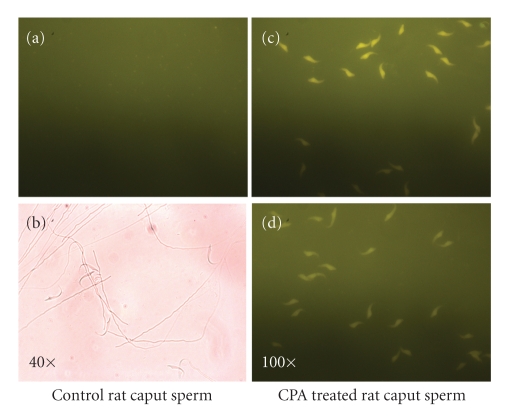
(a) Spermatozoa taken from untreated rats that have not picked up the CMA3 chromatin stain viewed under fluorescent microscope at 100 X magnification at 500–610 nm excitation/fluorescence emission wavelength. (b) Spermatozoa of untreated rats viewed under phase contrast objective at 40 X magnification. (c)-(d) Spermatozoa taken from the caput epididymides of CPA-treated rats that have picked up the intense yellow CMA3 nuclear chromatin stain in GC-rich regions normally occupied by protamine. Spermatozoa were fixed in Carnoy's fixative and stained with CMA3 dye in McIlvaine's buffer. Staining was viewed under fluorescent microscope at 100 X magnification at 500–610 nm excitation/fluorescence emission wavelength.

These studies had brought out the importance of prolactin in maintaining testosterone homeostasis and fertility status of male rats. Although the significance of testosterone to male fertility is well documented, the studies of Aleem et al. (2008) demonstrated for the first time the consequences of inadequate levels of this hormone to fertility in male rats [61] see (Figure 5). They demonstrated that blocking the androgen receptors with cyproterone acetate (CPA), at a dose of 20 mg/Kg/d for 15 days, to reduce the bioavailability of testosterone, led to underprotamination of epididymal sperm chromatin during spermiogensis in sexually competent male rats (see Figure 5). Our recent studies using CMA3 fluorescent dye uptake assay have confirmed the earlier observations of Aleem et al. (2005) in male rats. Cyproterone acetate-treated spermatozoa from caput epididymides incorporated the CMA3 dye at the GC-rich regions lacking protamine whereas the control rat spermatozoa were unreactive (Figures 6(a)–6(d)) [62].

### 2.3. Hypophyseal Factors

**Figure 7:**
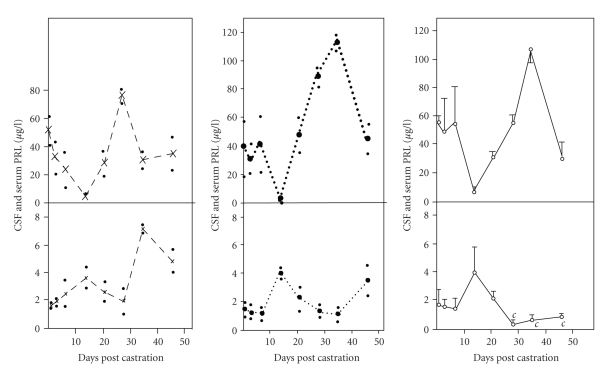
Comparative levels of PRL in the serum and CSF of male rats at different days following castration. Upper panels. Serum PRL levels. Lower panels. CSF PRL levels. Values are expressed in terms of NIADDK-rat-PRL-RP-3. “*c*” denotes significant difference at *P* < .05 level with respect to intact controls. Solid line with open circle (-∘-), dashed line with cross (–×–), and dotted line with closed circle (⋯•⋯) represent data from castrated, intact, and sham operated rats, respectively. At each day of castration, values are expressed as mean ± SEM of three or more determinations in duplicate of serum/CSF samples. In intact and sham-operated controls, each value is a mean of two determinants in duplicate of serum/CSF. Small dots (•) denote value of each determination. Vertical bars represent SEM. Prolactin was estimated by RIA in serum and CSF of normal and 1–46 day castrated rats [66].

In vitro studies on PHc system further led to the development of several in vivo models in our lab with a view to understanding the physiological relevance of the long and short feedback mechanisms involved in the regulation of pituitary hormones. Prolactin levels in the serum are known to be autoregulated through prolactin receptors expressed on the hypothalamic dopaminergic neurons [63]. The autoregulation of prolactin, referred to as the short feedback regulation, appears to be age-related and is purported to be mediated either through retrograde flow in the hypophyseal stalk or via cerebrospinal fluid (CSF) as a result of receptor-mediated uptake at the choroid plexuses [64, 65]. Balasinor et al. (1992) carried out studies in long-term castrated male rats and demonstrated that CSF is a vital link between the circulating prolactin and hypothalamic dopaminergic neurons that mediate its autoregulation [66]. The authors observed that bilateral castration of adult rats for 1, 3, 7, 14, 21, 28, 35, 46 days led to an increase in serum immunoactive prolactin levels but those in the CSF, removed from cisterna magna, displayed an inverse correlation (Figure 7). The turnover of hypothalamic dopamine decreased in castrated rats suggestive of a breakdown in the feedback mechanism in the dopaminergic neurons. Apparently either systemic bioactive prolactin was low or castration had led to a breakdown of receptor-mediated uptake mechanism at the choroid plexuses, as was suggested by low levels in CSF, with the result that prolactin was not reaching the dopaminergic neurons. Alternatively, dopaminergic neurons had become refractory to prolactin. It could be reasoned that estradiol of adrenal origin could have gained access to the dopaminergic neurons, downregulated dopamine turnover, and prevented prolactin autoregulation. In confirmation, a recent study by Aleem et al. (2006) suggested that administration of exogenous estradiol, at a dose of 100 ug/Kg/d, raised its systemic levels in male rats twofold within 10 days, concomitant with a fourfold increase in the secretion of prolactin [67]. Furthermore, Aleem et al. (2005), also observed that when the antiandrogen cyproterone acetate was administered to male rats at a dose of 20 mg/Kg/d for 15 days, a twofold decrease in the serum estradiol levels produced a twofold suppression of prolactin secretion [62].

## 3. Feedback Loops and Implications to Fertility Regulation in Male Rat

These in vitro and in vivo studies have confirmed that gonadal steroids, estradiol, and testosterone together constitute a long loop feedback mechanism for the regulation of prolactin secretion at the pituitary hypothalamic level. Whereas estradiol or long-term deprivation of testosterone stimulate the secretion of prolactin at the hypothalamic level, suggestive of a positive long feedback, testosterone-mediated suppression in vitro, and effects of castration in vivo indicate a negative feedback role at the pituitary.

The gaps in the information on the long loop feedback regulation of prolactin emerged from the studies carried out by Gill-Sharma et al. (2003) with the antipsychotic fluphenazine, a long-acting D2 dopamine receptor blocker, administered to male rats at a dose of 1–3 mg/Kg/d for 60 days to male rats [68]. The authors reported that fluphenazine competed with dopamine to block pituitary D2 receptors, leading to moderate hyperprolactinemia through a compensatory upregulation of hypothalamic tyrosine hydroxylase. Moderate increase in circulating prolactin led to a significant suppression of LH and FSH but not testosterone (Table 4). Grattan et al. (2007) reported that intracerebroventricular injection of prolactin in estrogen-primed, ovariectomized mice, led to the suppression of systemic LH [69]. The authors demonstrated that both estradiol and prolactin induced prolactin receptor in the gonadotropin releasing hormone (GnRH) neurons derived from transgenic mice expressing Green Fluorescent Protein (GFP) under the control of GnRH promoter (GnRH-GFP) and implicated it in LH suppression. More recently, Anderson et al. (2008) have reported that chronic hyperprolactinemia induced by dopamine antagonist, Sulpiride, suppressed LH pulse frequency in estradiol-primed, ovariectomized rat [70]. The authors further observed that estradiol augments the effects of Sulpiride-induced prolactin on the expression of prolactin receptor and associated inhibitory signaling molecules viz. STAT-5, SOCS-1 and 3, and CIS in the hypothalamic GnRH neurons of estrogen-primed ovariectomized rat. The authors suggested that prolactin inhibited the LH pulses in vivo since prolactin-induced suppression of GnRH has already been reported in immortalized GT1 cell lines derived from murine GnRH neurons [71]. Such an effect of hyperprolactinemia on LH had also been reported by Bartke et al. (1977) [72]. The chromatin of fluphenazine-treated epididymal spermatozoa was susceptible to denaturation in vitro. The fertilizing potential of the poor quality of spermatozoa produced by the fluphenazine-treated rats was severely compromised as indicated by reduction in litters sired. Sires displayed a tendency to mate later than their untreated counterparts indicative of an effect on reproductive behaviour which was considered to be due to direct effects of moderately high levels of prolactin on central incertohypothalamic neurons (IHDA). The authors averred that the expected inhibition of testosterone did not occur due to a direct effect of prolactin on its synthesis in the Leydig cells [68, 73]. In support of the earlier observations suggestive of feedback repression by testosterone, the in vivo study suggested that prolactin could autoregulate its own secretion by upregulating the synthesis of testosterone. It is tempting to speculate, on the basis of the observations of Jeyaraj et al. (2005) and Aleem et al. (2006) that prolactin could also be playing a compensatory role in maintaining circulating testosterone levels after sequestration of its circadian peak by estradiol-induced androgen binding protein in the Sertoli cells of rat testis [67, 74]. Thus the autoregulatory role of prolactin appears to be exerted both at the hypothalamic and testicular levels. It can be hypothesized on the basis of these results that the effects of moderate hyperprolactinemia on fertility can be reversed by autoregulation through the long and short feedback loops.

**Figure 8:**
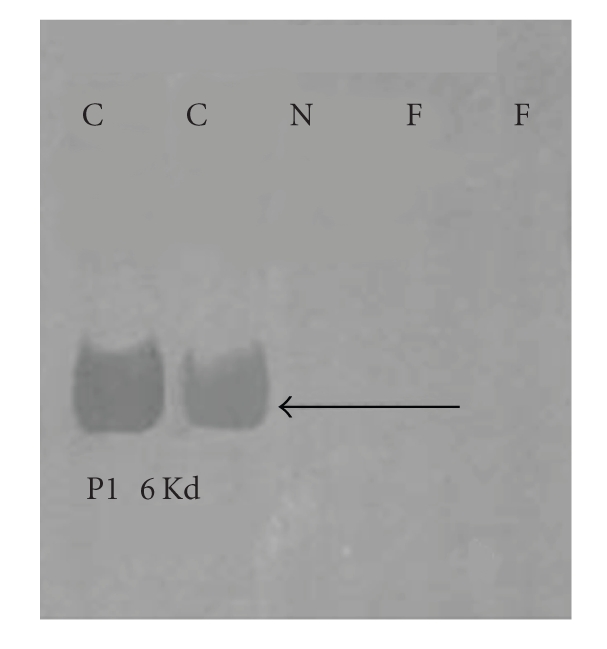
Western blots of protamine 1 in epididymal sperm of sexually mature rats depicting the presence of 6 Kd protamine 1 bands in control and absence in drug-treated rats. F: Fluphenazine; C: Control; N: negative control. Basic proteins were extracted in HCl, differentially extracted in trichloroacetic acid, analysed on 15% acid urea PAGE. Western blotting was done with 1Hup 1N monoclonal antibody provided by Dr Rod Balhorn [61].

**Figure 9:**
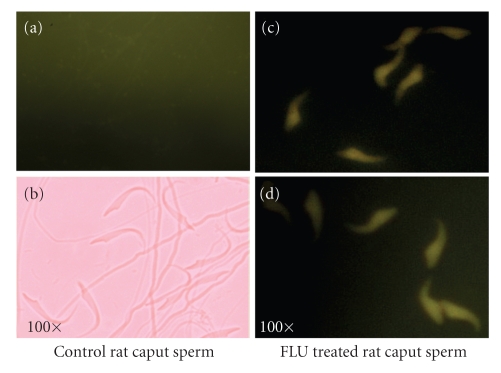
(a) Spermatozoa taken from untreated rats that have not picked up the nuclear chromatin stain viewed under fluorescent microscope at 100 X magnification at 500–610 nm excitation/fluorescence emission wavelength. (b) Spermatozoa of untreated rats that have not picked up the stain viewed under phase contrast objective at 100 X magnification. (c)-(d) Spermatozoa taken from the caput epididymides of fluphenazine-treated rats that have picked up the intense yellow CMA3 nuclear chromatin stain in the GC-rich regions occupied normally by protamine. Spermatozoa were fixed in Carnoy's fixative and stained with CMA3 dye in McIlvaine's buffer. Staining was viewed under fluorescent microscope at 100 X magnification at 500–610 nm excitation/fluorescence emission wavelength.

However, further studies by Aleem at al. (2005) with fluphenazine in male rats did not support the corrective hypothesis on fertility. They observed that in spite of the compensatory feedback upregulation of testosterone by prolactin, the FSH levels remained significantly suppressed [72, 75]. The effects of FSH inhibition could be observed on the mechanism of sperm chromatin condensation during spermiogenesis in the testis. The authors observed that levels of several key proteins involved in chromatin condensation viz. cyclic adenosyl monophosphate regulatory element modulator (CREM*ι*), transition proteins, and protamine 1 were suppressed in the testis. The epididymal spermatozoa of treated male rats lacked protamination and were loosely packaged (see Figure 8). Recent in vitro studies carried out in our lab used fluorescent CMA3 dye uptake assay to demonstrate the protamination status of spermatozoa. It was observed that spermatozoa taken from the caput epididymides of untreated male rats did not incorporate the CMA3 dye in the chromatin (see Figures 9(a)-9(b)). The uptake of intense yellow CMA3 stain in the GC-rich regions of fluphenazine-treated epididymal sperm chromatin confirmed the absence of protamination in these regions where protamine normally binds (see Figures 9(c)-9(d)). Thus, in spite of operational long and short feedback mechanisms, moderate hyperprolactinemia was observed to lead to production of poor quality spermatozoa.

## 4. Feedback Loops and Fertility Regulation in Men

Although it is not always possible to extrapolate the results of animal studies to humans, studies carried out in our lab by Juneja et al. (1991) suggested that feedback regulation by gonadal steroids might be a conserved phenomenon [76]. The authors determined the pattern of circadian release of reproductive hormones from the hourly blood samples drawn from normal young men (Table 5). Their studies suggested that the physiological relationship apparent between prolactin and testosterone in male rats was also reflected in human males. The authors observed an inverse correlation between the acrophase of circadian release of bioactive prolactin with nadir of LH and a direct correlation with that of the acrophase of testosterone release in men. The temporal coupling of prolactin release between 20 and 23 hours followed by suppression of bioactive LH between 24 and 2 hours and peak release of testosterone between 1 and 5 hours was suggestive of a direct role for prolactin in entraining testosterone release essential for male behaviour while LH release was being restrained at the level of hypothalamic GnRH and opiatergic neurons and adenohypophyseal gonadotrophs by both the hormones. Interestingly, peak release of testosterone appeared to have led to suppression of prolactin between 9 and 11 hours concomitantly with that of testosterone itself. Testosterone nadir coincided with a surge of bioactive LH between 9 and 11 hours suggesting that the combined feedback inhibition by the two hormones would have been released to allow autopriming of the GnRH receptors on the gonadotrophs and synthesis of LH receptors on the Leydig cells. The suppression of testosterone probably led to a surge in estradiol between 15 and 18 hours, presumably due to activation of P450 aromatase in the Leydig cells. Estradiol was then observed to entrain the prolactin peak between 20 and 23 hours. That the prolactin peak is of pituitary and not extrapituitary origin could be averred from the fact that only hypophyseal prolactin gene has estrogen response elements in its promoter [60]. Their studies suggested that the temporal coupling between prolactin and testosterone could signify a functional relationship that may be playing an important role in the regulation of human male fertility. Although differences have been reported between the prolactinergic mechanisms between rat and human, particularly in terms of extrapituitary sites of synthesis, which would argue against extrapolations, this particular study led to the tentative conclusion that gonadal steroids, testosterone, and estradiol could constitute a conserved physiological long feedback mechanism between gonads and adenohypophysis which likely ensures the circadian release of prolactin in systemic circulation, and maintains male fertility and libido. This view is supported by the fact that both rat and human hypophyseal prolactin genes share estrogen response elements in their promoters [77].

## 5. Conclusions

The results of our in vitro and in vivo studies suggest that prolactin is a crucial hormone for the regulation of male fertility with a defined ontogenetic pattern of development of the hypothalamic releasing and inhibitory factors that determine its level in circulation at adulthood. Adult hypothalami contain prolactin-responsive dopaminergic neurons for autoregulation as well as stimulation of opiatergic neurons involved in the inhibition of GnRH neurons. In addition to the hypothalamic regulatory factors, adenohyphyseal lactotrophs at adulthood express a testosterone-responsive inhibitory mechanism for prolactin synthesis and estrogen-dependent stimulatory mechanisms for prolactin secretion, whereas Leydig cells in the testis express a prolactin-dependent regulatory mechanism for testosterone synthesis. The prolactin responsive mechanisms constitute the biological substrates for the pituitary-hypothalamo-gonadal feedback system in the male mammals, which relies on the gonadal steroids, testosterone and estradiol for long-loop, and prolactin for short-loop feedback mechanisms. These mechanisms would operate to contain the adverse effects of reproductive stress and ensure that serum levels of prolactin remain within the physiological range, because even mild-to-moderate hyperprolactinemia, if allowed to become chronic, affects the quality of mature spermatozoa and their fertilizing potential. Hyperprolactinemia also affects reproductive behaviour in spite of normal testosterone levels. Importantly, whereas the effects of acute hyperprolactinemia appear to be mediated via testosterone inhibition, those due to moderate hyperprolactinemia would be a consequence of FSH deficits.

## Figures and Tables

**Table 1:** Release of Prolactin by different pituitary constructs in vitro.

System	Whole pituitary		Whole pituitary		Pituitary-hypothalamus	
			Plus hypothalamus		complex	
Incubation(h)	Total^a^	Per h^b^	Total^a^	Per h^b^	Total^a^	Per h^b^
2	371.3 ± 42.9	185.6 ± 21.5	281.4 ± 22.9	140.7 ± 11.5	227.9 ± 28.9^b^	113.9 ± 14.4
4	360.2 ± 34.6	90 ± 8.7**	294.9 ± 31.1	73.7 ± 7.8*	345.4 ± 21.9**	86.4 ± 5.5
6	437.8 ± 56.6	72.5 ± 9.4*	401.9 ± 24.3**	67 ± 4.1*	270.4 ± 30.7^c^	45 ± 5.1*

Pituitary constructs were incubated in DMEM at 37°C for 4 hours, in replicates of 10, at each time interval. Prolactin was assayed in spent medium by RIA. Values are expressed as mean ± SEM. ^a^ng Prl (NIAMDD-RP-2); *P < .001, **P < .01, with respect to levels at 2 hours; ^b^ *P* < .02, ^c^ *P* < .05, with respect to corresponding pituitary levels [27].

**Table 2:** Effects of reserpine on prolactin secretion by different pituitary constructs in response to dopamine agonists and antagonists in vitro.

Additions	Control			Reserpine 1.5 mg/kg/d		
	In 25% Ascorbic acid			in 25% ascorbic acid		
(in vitro)	PI	P-N	PHc	PI	P-N	PHc
Ascorbic acid	429 ± 14.8	702 ± 8.54^c^	316 ± 7.59	126 ± 1.9^b^	126 ± 3.8^b^	167 ± 4.4^bc^
(0.05%)						
Dopamine	344 ± 12.34	219 ± 11.39^a^	124 ± 2.85^a^	50 ± 1.27^ab^	71 ± 3.8^ab^	59 ± 1.9^ab^
10^−7^ M						
Bromocriptine	317 ± 10.12	339 ± 5.38^a^	161 ± 5.06^a^	46 ± 2.53^ab^	56 ± 3.8^ab^	50 ± 1.9^ab^
10^−7^ M						
Apomorphine	364 ± 5.06	108 ± 5.38^a^	108 ± 11.71^a^	40 ± 1.27^ab^	60 ± 2.53^ab^	30 ± 1.27^b^
8 × 10^−7^ M						
Perphenazine	393 ± 10.12	694 ± 3.8^c^	466 ± 5.7*	140 ± 3.8	140 ± 6.96	330 ± 5.7*
5 × 10^−7^ M						

Reserpine was dissolved in 25% ascorbic acid and administered s.c to adult male rats for 46 hours. Control group rats received vehicle alone. Different pituitary constructs were dissected out in DMEM and incubated at 37°C for 4 hours, in replicates of 10, at each time interval. Prolactin was assayed in spent medium by RIA. Values are expressed as mean (ng NIDDK-RAT-PRL-RP-3^−ml^ incubation medium) ± SEM. ^a^significant difference with respect to 0.05% ascorbic acid (*P* ≤ .05). ^b^significant difference with respect to 25% ascorbic acid control group (*P* ≤ .05). ^c^significant difference with respect to PI and PHc of the same group (*P* ≤ .05). *significant difference with respect to PI and P-N of the same group (*P* ≤ .05) [33].

**Table 3:** Effects of estradiol 17*β* on serum hormones in adult male rat.

Estradiol 17*β*	Plasma levels (ng/mL)			
(*μ*g/kg/d)	LH	FSH	PRL	T
0	0.85 ± 0.09	6.16 ± 0.32	122.37 ± 6.17	2.17 ± 0.31
0.1	0.89 ± 0.108	5.58 ± 0.498	115.80 ± 4.03	1.50 ± 0.17
10	0.54 ± 0.109*	3.49 ± 0.38*	120.60 ± 4.70	1.44 ± 0.19
100	0.43 ± 0.09*	2.38 ± 0.24*	255.50 ± 17.9*	0.72 ± 0.04*
200	0.31 ± 0.08*	2.67 ± 0.17*	306.00 ± 22.27*	0.84 ± 0.09*
300	0.19 ± 0.05*	2.65 ± 0.13*	370.00 ± 13.60*	0.89 ± 0.06*
400	0.17 ± 0.03*	2.48 ± 0.56*	364.30 ± 38.07*	0.83 ± 0.10*
1000	0.36 ± 0.096*	2.98 ± 0.18*	465.00 ± 51.19*	0.63 ± 0.097*

Estradiol 17*β* was dissolved in saline by sonication and injected s.c. Male rats were mated at 60 days of treatment and serum hormones analyzed by RIA. All values are expressed as mean ± SEM. Dose “0” represents control values. *denotes significance at *P* ≤ 0.5 as compared to controls [59].

**Table 4:** Effects of fluphenazine decanoate on fertility parameters and serum hormones in adult male rat.

Affected	Fluphenazine treatment (mg/kg/d) for 60 days			
parameters	0	1	2	3
Prolactin, ng/ml	18.99 ± 1.82	76.80 ± 11.1*	82.53 ± 10.6*	139.30 ± 25.1*
LH, ng/ml	1.13 ± 0.26	0.23 ± 0.04*	0.44 ± 0.05*	0.54 ± 0.05*
FSH, ng/ml	7.72 ± 0.26	5.43 ± 0.44*	6.08 ± 0.31*	6.00 ± 0.22*
Testosterone, ng/ml	2.60 ± 0.14	2.17 ± 0.17	2.40 ± 0.20	2.32 ± 0.24
Estradiol, ng/ml	82.00 ± 9.51	114.2 ± 21.3	70.61 ± 8.14	58.19 ± 10.4
Sperm count, million/cauda	63.8 ± 3.89	54.3 ± 4.18	52.8 ± 3.53	59 ± 6.68
Potency, %	100 ± 00	83.3 ± 11.24	100 ± 00	66.7 ± 14.21*
Fecundity, %	100 ± 00	66.7 ± 21.09	83.3 ± 16.67	50 ± 22.36
Implantation sites, per rat	11.3 ± 1.16	6.7 ± 1.67	5.1 ± 1.54*	5.4 ± 1.69*
Litters sired, per female	10.3 ± 1.17	6.4 ± 1.6	4.8 ± 1.49*	5.7 ± 1.73*
Fertility index, per rat	0.9 ± 0.08	0.6 ± 0.14	0.5 ± 0.15*	0.5 ± 0.15*

Fluphenazine was dissolved in sesame oil and injected s.c. Male rats were mated at 60 days of treatment and serum hormones analyzed by RIA. Uterine horns of mated females were exposed to count implantation sites, ovaries for corpora lutea. All values are expressed as mean ± SEM. Dose “0” represents control values. *denotes significance at *P* ≤ .05 as compared to controls. Potency = % mated females; fecundity = % male rats siring at least one viable pup; fertility index = ratio of implantation sites to corpora lutea; litter size = number of pups per female [68].

**Table 5:** Diurnal hormone variations in adult men.

Hormone/mL	Mean	Circadian	Acrophase (h)	Nadir (h)
	concentration	amplitude	(range)	(range)
T, ng	3.56 ± 1.94*	3.1 ± 0.8*	1–5	9–12
E_2_, pg	55.9 ± 26.9*	38 ± 7.2*	15–18	24–2
BLH, mIU	55.4 ± 6.25*	14.4 ± 2.2*	9–12	24–2
ILH, mIU	7.4 ± 1.7*	3 ± 0.6*	1–5	15–17
BPRL, ng	4.9 ± 1.7*	2.6 ± 0.4*	20–23	9–11
IPRL, ng	11.6 ± 2.0*	4.1 ± 0.8*	23–4	11–15

Hormones in the hourly blood samples of healthy men were assayed by RIA. Hormone concentration and circadian amplitudes are expressed per milliliter plasma. * = SD. T = testosterone; ILH = immunoreactive LH (NIADDK-LER-907); BLH = bioactive LH (WHO 2nd IRP 78/549); BPRL = bioactive PRL (NIAMDD-hPRL-I-6); IPRL = immunoactive PRL (NIAMDD-hPRL-I-6); E_2_ = 17-*β*-estradiol [76].
